# High ice nucleation activity located in blueberry stem bark is linked to primary freeze initiation and adaptive freezing behaviour of the bark

**DOI:** 10.1093/aobpla/plu044

**Published:** 2014-07-31

**Authors:** Tadashi Kishimoto, Hideyuki Yamazaki, Atsushi Saruwatari, Hiroki Murakawa, Yoshihiko Sekozawa, Kazuyuki Kuchitsu, William S. Price, Masaya Ishikawa

**Affiliations:** 1 Division of Plant Sciences, National Institute of Agrobiological Sciences, Kannondai 2-1-2, Tsukuba, Ibaraki 305-8602, Japan; 2 Present address: International Patent Organism Depository, National Institute of Technology and Evaluation, Kisarazu, Chiba 292-0818, Japan; 3 Applied Biological Science, Graduate School of Science and Technology, Tokyo University of Science, Yamazaki 2641, Noda, Chiba 278-8510, Japan; 4 Faculty of Life and Environmental Sciences, University of Tsukuba, Tennodai 1-1-1, Tsukuba 305-8572, Japan; 5 Nanoscale Organisation and Dynamics Group, University of Western Sydney, Penrith, NSW 2751, Australia

**Keywords:** Blueberry, cold hardiness, extracellular freezing, freezing tolerance, ice nucleation, infra-red thermography, *Vaccinium ashei*, *Vaccinium corymbosum*

## Abstract

Controlled ice nucleation is an important mechanism in cold-hardy plant tissues for avoiding excessive supercooling of the protoplasm, for inducing extracellular freezing and/or for accommodating ice crystals in specific tissues. To understand its nature, it is necessary to characterize the ice nucleation activity (INA), defined as the ability of a tissue to induce heterogeneous ice nucleation. Few studies have addressed the precise localization of INA in wintering plant tissues in respect of its function. For this purpose, we recently revised a test tube INA assay and examined INA in various tissues of over 600 species. Extremely high levels of INA (−1 to −4 °C) in two wintering blueberry cultivars of contrasting freezing tolerance were found. Their INA was much greater than in other cold-hardy species and was found to be evenly distributed along the stems of the current year's growth. Concentrations of active ice nuclei in the stem were estimated from quantitative analyses. Stem INA was localized mainly in the bark while the xylem and pith had much lower INA. Bark INA was located mostly in the cell wall fraction (cell walls and intercellular structural components). Intracellular fractions had much less INA. Some cultivar differences were identified. The results corresponded closely with the intrinsic freezing behaviour (extracellular freezing) of the bark, icicle accumulation in the bark and initial ice nucleation in the stem under dry surface conditions. Stem INA was resistant to various antimicrobial treatments. These properties and specific localization imply that high INA in blueberry stems is of intrinsic origin and contributes to the spontaneous initiation of freezing in extracellular spaces of the bark by acting as a subfreezing temperature sensor.

## Abstract (precis)

One may have seen wintering rosette leaves totally frozen and wilted in the early morning but recover during the daytime. How can cold hardy plants survive freezing of the tissues, unlike animal tissues? Cold hardy plants seem to have evolved various strategies. One example is extracellular freezing, where icicles primarily form in intercellular spaces whilst the cells are dehydrated, yet the underlying mechanisms remain unclear. In this study, using blueberry stems, we found high ice nucleation activity specifically localized in the cell wall fraction of bark tissues. This activity likely contributes to the primary and spontaneous initiation of freezing in the intercellular spaces of the bark to successfully perform extracellular freezing.

## Introduction

Cold-hardy woody plant tissues display diverse but controlled freezing behaviour under subfreezing temperatures. These include extracellular freezing (e.g. in bark), deep supercooling (e.g. in xylem ray parenchyma) and extra-organ freezing (e.g. flower buds and leaf buds) (Ishikawa and Sakai 1982; Sakai and Larcher 1987). These types of freezing are specific to species and tissues and are important determinants of their degree of cold hardiness and survival (Ishikawa *et al.* 2000, 2009). Yet, mechanisms that allow tissues to perform their specific intrinsic freezing behaviour remain unanswered. These may include controlled management of ice nucleation and propagation, water flow, stabilization of supercooling, inhibition of icicle growth/sublimation by antifreeze, recrystallization inhibition and morphological or physical barriers (Ishikawa *et al.* 2009). Among these, ice nucleation is the primary event when the plant encounters subfreezing temperatures. It is considered to be important for initiating and regulating freezing behaviour such as extracellular freezing and extra-organ freezing. In extracellular freezing, initiation of ice formation outside the cell is a prerequisite. This generates a driving force for withdrawing cellular water to the apoplast (extracellular space) according to the chemical potential difference between aqueous solution and ice. Moreover, icicles tend to form in a particular space that depends on the tissue or organ (Wiegand 1906; Pearce 2001; Wisniewski *et al.* 2009). In extra-organ freezing, ice formation occurs only on specific tissues (e.g. bud scales) and generates an ice sink. This allows slow withdrawal of water from the supercooled tissues (e.g. florets) to the ice sink and enhances the supercooling ability of the tissues (Quamme 1978; Ishikawa and Sakai 1981, 1982, 1985; Price *et al.* 1997). However, the mechanisms for controlled initiation of freezing (ice nucleation) in wintering cold-hardy plant tissues remain obscure. Determination of ice nucleation activity (INA) in tissues (i.e. the ability to cause heterogeneous ice nucleation, hereafter referred to as INA) and identification of responsible ice nucleators will help address this long-unanswered question.

Historically, efforts to identify ice nucleators in plants have been made mainly with respect to late spring or early autumnal frost injury in summer crops and fruit trees (reviewed in Ashworth and Kieft 1995; Hirano and Upper 1995). Most freezing-sensitive summer crops such as potato, maize and beans lack effective ice nuclei active at warmer than −6 °C (Marcellos and Single 1979) and epiphytic ice-nucleating bacteria such as some strains of *Pseudomonas syringae* and *Erwinia herbicola* are considered responsible for the lethal freezing of plants at warm subzero temperatures (Lindow 1983). In fruit trees, spring frosts cause much damage to developing flowers (Rodrigo 2000) and ice nucleation of fruit tree shoots in this season has been attributed largely to non-bacterial sources of INA, although epiphytic bacterial INA may also be involved (Ashworth and Kieft 1995).

Ice nucleation activity of bacterial origin has been studied extensively and characterized in detail (reviewed in Hirano and Upper 1995; Upper and Vali 1995). A high INA has also been found in fungi (*Fusarium*) (reviewed in Ashworth and Kieft 1995). The INA of these organisms was considered to be proteinaceous (reviewed in Ashworth and Kieft 1995; Fall and Wolber 1995), and the gene responsible for the ice nucleation-active protein was identified in ice-nucleating bacteria based on the mutant analysis (reviewed in Warren 1995).

In contrast, attempts to isolate the substances responsible for plant intrinsic INA have been unsuccessful as they often resulted in a loss of activity or because solubilization proved difficult (Pearce 2001). This allowed some to believe that plant ice nucleators are mostly of epiphytic microbial origin. However, woody plants have INA with different properties from those of microbial origin (Ashworth and Kieft 1995). The INA of afroalpine *Lobelia telekii* (−4 °C) was boiling stable and likely a carbohydrate in nature (Krog *et al.* 1979; Embuscado *et al.* 1996). The INA from flower bud scales of *Rhododendron japonicum* (−5 to −6 °C) was resistant to autoclaving (Ishikawa *et al.* 2000). The INA in *Prunus* wood (−2 to −6 °C) was resistant to protein denaturing and degrading treatments and had different sensitivity to heating and chemicals than bacterial INA (Ashworth and Davis 1984; Gross *et al.* 1988). The INA of winter rye leaves (−7 to −8 °C) appeared to involve elements of protein, phospholipids and carbohydrate (Brush *et al.* 1994). All these studies were based primarily on differential sensitivity of the specimens to various treatments compared with bacterial INA (Ashworth and Kieft 1995), and the identity of plant INA compounds remains ambiguous (Wisniewski *et al.* 2009). The history of bacterial ice nuclei research reveals the importance of finding good materials with high INA and abundant availability for further studies.

To resume and advance INA research, we developed a highly reproducible assay for determining the INA of plant tissues by revising conventional test tube nucleation assays and using the new assay, we surveyed plant tissues of over 600 species for INA (e.g. Sekozawa *et al.* 2002; Ueda *et al.* 2002; Ishikawa 2014). Extremely high INA (−1 to −4 °C) was found in the stems of wintering blueberry (Kishimoto *et al.* 2014), much higher than other cold-hardy plants such as peach (−6 °C) or *Rhododendron* (−5 to −6 °C) investigated by others. Blueberry stems facilitate INA studies by being available in large quantities and having high INA. To help reveal their origin, development, spatial and temporal distribution, turnover, occurrence and roles in adaptation to freezing temperatures, extensive studies are necessary (reviewed by Wisniewski *et al.* 2009).

The present study characterizes the high INA in blueberry stems with emphasis on the spatial localization. We reveal in which organ, tissue and cellular fractions the INA are localized, and the concentration of active ice nuclei in stems and evaluate the effect of antimicrobial treatments and vacuum infiltration. We also considered the functional roles of the high INA, i.e. how it relates to freezing behaviour, ice accumulation in the tissue and ice nucleation.

## Methods

### Plant material

Unless otherwise specified, current-year stems were randomly collected in winter months from ≥20-year-old trees (∼4 m tall) of high-bush blueberry (*Vaccinium corymbosum* cv. Weymouth) and rabbit eye (*Vaccinium ashei* cv., Woodard) at the nurseries of Tsukuba University and our institute (50 km northeast of Tokyo), Ibaraki, Japan,. These two cultivars represent the two major species of blueberry grown in this area and exhibit contrasting levels of cold hardiness (LT_50_ for Weymouth and Woodard in mid-winter: −25 and −16 °C, respectively). The samples were cooled on ice and used immediately for experiments unless otherwise noted.

### Differential thermal analysis

Current-year stems (2-cm-long internodes) of blueberry were cooled at 5 °C h^−1^ from 20 to −40 °C in a programmable deep freezer (Model FPR-120S, Fuji Ika Sangyo Co. Ltd). Exothermic events were detected with a copper-constantan thermocouple attached to the stem surface and amplified 40–100 times prior to recording (Ishikawa and Sakai 1981). At least six replicates were performed for each determination.

### Visual observation of stems at subfreezing temperatures

Blueberry stems (∼20 cm long) collected in late December were enclosed in polyethylene bags in an insulated cool box and cooled at −2 °C h^−1^ to −10 °C in a programmable freezer (Model FPR-120S, Fuji Ika Sangyo Co. Ltd), then transferred to a cold room (−10 °C) without thawing and stored for 3–5 days before the localization of ice was determined visually in stem tissues dissected under a binocular microscope.

### Determination of INA

**Figure 1.**
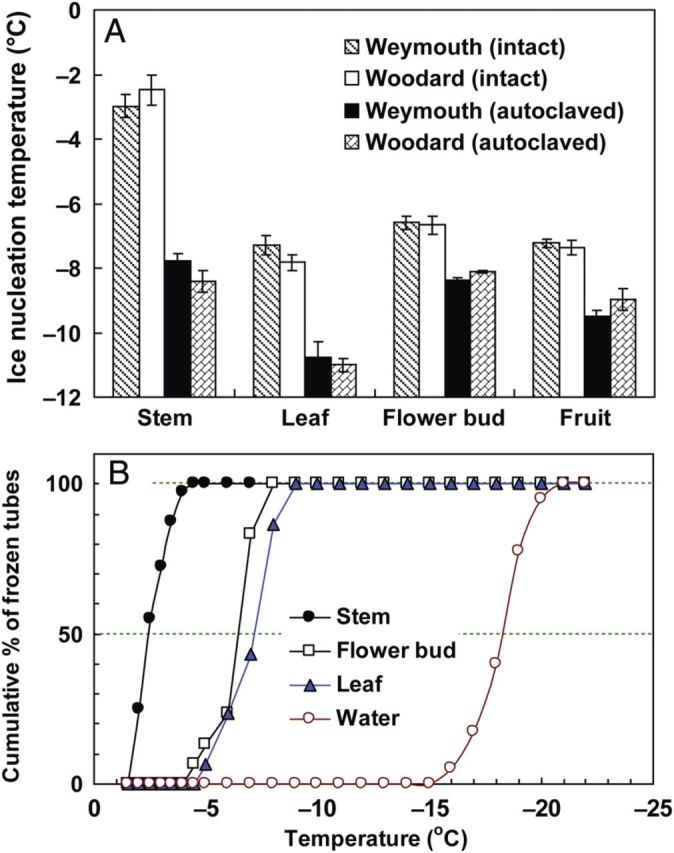
Distribution of INA as shown by ice nucleation temperatures (INTs) in various terrestrial organs on current-year shoots of high-bush (cv. Weymouth) and rabbit-eye (cv. Woodard) blueberry trees and their tolerance to autoclaving for 15 min at 121 °C (A). The data are the mean ± SE (*n* = 3–6) of the median INT determined with stems (September to January), leaves (August to November), flower buds (August to January for Weymouth and October to January for Woodard) and fruits (June to July for Weymouth and August to September for Woodard) using the test tube method (40 tubes, each containing 0.5 mL sterilized Milli-Q water and the sample). A typical example of INT determination of November samples (Weymouth) was also shown as increases in the cumulative number of frozen tubes (B). The median (50 %) INT was either estimated from the graph or calculated from Eq. (1). Methods of the INA assay, sample processing and autoclaving are detailed in the Methods section.

The INA of various organs (current-year stem, leaf, flower bud and fruit) was determined by measuring ice nucleation temperature (INT) of the samples using a modified test tube nucleation method. Stems and leaf blades (petioles and main midribs were not included) were cut into 7.5-mm-long pieces or 7.5 mm^2^, respectively, and used for the assays. For flower buds, a single whole organ, and for fruits, a half-cut piece or single organ (to fit the tube diameter), was placed in 0.5 mL Milli-Q water in TPX test tubes (40 replicates for a sample) sealed with transparent polyester film. All equipment in contact with the Milli-Q water in the assay tubes was pre-autoclaved at 121 °C for 20 min, including the tubes, lids and water. Tissue excision and placement into the tubes were done using clean knives and forceps in a laminar flow chamber to avoid contamination from air-borne ice nuclei of microbial or other origin. These procedures eliminated nucleation above −15 °C of the control tubes containing 0.5 mL Milli-Q water (without samples). Their average median INT was –18.5 at ∼ −19.2 °C (Fig. 1B). In preliminary experiments, stem samples were surface-sterilized with 0.1 % sodium hypochlorite for 30 min prior to INA determination. This was found to affect the stem INA only marginally and this process was usually omitted. The tubes were cooled in a stepwise manner in a precision-controlled bath (coolant ∼40 % v/v ethylene glycol) from 0 to −20 °C at 1 °C decrements (Kishimoto *et al.* 2014). The bath temperature was regulated using a proportional-integral-derivative (PID) controller and a Pt temperature sensor to minimize temperature overshooting and space- and time-wise errors within 0.1 °C. Cooling 1 °C from one step to another took ∼5 min. The tubes were maintained at each temperature for 20 min before counting the number of frozen tubes. The temperature of the sample inside the tubes reached the bath temperature within 1 min and temperature errors were within 0.1 °C. Frozen or unfrozen tubes were easily recognized by viewing the tube content through the transparent lid using backlighting introduced into the bath with an optical fibre (Kishimoto *et al.* 2014). Longer incubation periods did not increase the number of frozen tubes significantly as most freezing events were found to occur in the first 10–15 min if the incubation temperature was between 0 and –12 °C. Freezing events tended to be more affected by prolonged incubation at each temperature when the temperature was lower than –12 °C. To minimize the duration of the experiments and make the experimental data comparable, we also adopted a 20-min incubation period for temperatures below –12 °C. We obtained the highly reproducible data of INT using this method as shown by the small SEs in Tables 1 and 2 and Figs 4, 6B and 9.

**Table 1.** Effect of incubation with antibiotics, NaClO and HgCl_2_ on the INT of blueberry stems collected in January.

Treatments	Median INT (°C)	
	Weymouth	Woodard
Intact stem (control)	−4.2 ± 0.2	−4.3 ± 0.1
NaClO	−4.4 ± 0.3	−4.7 ± 0.3
HgCl_2_	−4.0 ± 0.2	−4.4 ± 0.1
Doxycycline	−3.8 ± 0.4	−4.4 ± 0.2
Kanamycin	−4.0 ± 0.1	−3.8 ± 0.3
Rifampicin	−4.3 ± 0.1	−4.7 ± 0.3
Penicillin G	−4.3 ± 0.1	−4.9 ± 0.3
Oxytetracycline	−4.4 ± 0.1	−4.4 ± 0.2

**Table 2.** Effects of imbibition in *P. syringae* solution and autoclaving (121 °C for 15 min) on the median INT of current-year blueberry stems (cv. Weymouth and Woodard) collected in November and January. The data presented are the mean ± SE of four experiments, each of which determined the median INT of 40 test tubes containing 0.5 mL sterilized Milli-Q water and stem segments (7.5 mm long). *Pseudomonas syringae* (the last line of the table) indicates the median INT of tubes containing 0.5 mL Snomax^®^ solution (containing a gamma ray-sterilized ice nucleation-active strain of *P. syringae*) before and after autoclaving. Similarly, +*P. syringae* indicates the median INT of tubes containing stem segments and Snomax^®^ solution.

Samples	Months	Treatments	Intact median INT (°C)	Autoclaved median INT (°C)
Weymouth stem	November	Nil	−2.3 ± 0.1	−7.3 ± 0.1
	November	+*P. syringae*	−2.3 ± 0.0	−7.4 ± 0.5
	January	Nil	−4.2 ± 0.3	−9.2 ± 0.1
	January	+*P. syringae*	−3.6 ± 0.0	−9.1 ± 0.2
Woodard stem	November	Nil	−1.8 ± 0.0	−9.2 ± 0.1
	November	+*P. syringae*	−1.8 ± 0.0	−9.1 ± 0.1
	January	Nil	−4.3 ± 0.2	−9.4 ± 0.2
	January	+*P. syringae*	−3.6 ± 0.1	−9.8 ± 0.1
*P. syringae*			−3.7 ± 0.0	−19.2 ± 0.4

Ice nucleation activity was expressed as the median INT (median *T*_INT_) at which 50 % of the tubes were frozen. This was either extrapolated from the graph or calculated from the following equation:

(1) $$\text{Median}\;\;T_{\mathrm{INT}}=T_{1}+(T_{2}-T_{1})({\text{2}}^{-1}n-F_{1})(F_{2}-F_{1}{)}^{-1},$$

where *F*_1_ and *F*_2_ are the cumulative number of frozen tubes at temperature *T*_1_ and neighbouring decremental temperature *T*_2_, respectively, and are just below and above 50 % of the total number of tubes (*n*), respectively. When necessary, the distribution of the INT was shown by plotting the cumulative number of tubes frozen or the associated histogram. Unless otherwise specified, the experiments were replicated four times and the mean ± SE of the four median INT was shown.

In some experiments, the length (mass) of the stem piece placed in a tube was altered from 0.83 to 23 mm (weighing 1.4–39 mg for Woodard and 3.5–98 mg for Weymouth) instead of standard 7.5 mm stem pieces (∼13 mg for Woodard and 32 mg for Weymouth) to check the effect of sample mass on the INT determined using the test tube method (Fig. 4). From the raw data of these nucleation assays, it is possible to estimate the cumulative ice nuclei concentration *K*(*T*), which is an indication of the numbers of ice nuclei that acted at all temperatures warmer than *T* in the unit volume of water (Vali 1971):

(2) $$K(T)=-\text{LN}(\;f)V^{-1}(\text{expressed}\;\;\text{as}\;\;\text{ice}\;\;\text{nuclei,}\;\;{\text{mL}}^{-1}),$$

where *f* is the fraction of tubes unfrozen at temperature *T* and *V* is the volume of water in the assay tubes. *f* can be calculated from (*n*–*F_T_*) *n*^–1^, where *F_T_* is the cumulative number of tubes frozen at temperature *T* and *n* is the total number of tubes. Since the stem mass (*M*) in *V* are known, it is possible to calculate *K*′(*T*), the cumulative concentration (per unit mass of the sample) of ice nuclei that acted at all temperatures warmer than *T* (Vali 1995):

(3) $$K^{\prime }(T)=-\text{LN}(\;f)M^{-1}(\text{expressed}\;\;\text{as}\;\;\text{ice}\;\;\text{nuclei,}\;\;{\text{g}}^{-1})$$

Plotting the cumulative ice nuclei concentrations active (effectively nucleating) at a wide range of temperatures gives a picture of the cumulative ice nuclei spectrum (e.g. Fig. 5). Serial reduction in the stem mass results in a serial reduction in INT, which enables the entire ice nuclei spectrum of stem samples to be obtained. This was done in analogy with the serial dilutions of microbial suspensions used in studies on ice-nucleating bacteria and fungi. *K*′(*T*) is shown in the form of Log_10_ *K′*(*T*) in Fig. 5.

When necessary, a dilute solution of the bacterial ice nucleator, Snomax^®^ (York Snow Co., New York, NY, USA) was prepared and used as a positive control of ice nucleation assays. Snomax^®^ comprised freeze-dried ice-nucleating bacteria, *P. syringae*, sterilized by gamma ray irradiation. In some cases, INT was determined with stem segments placed in 0.5 mL of Snomax^®^ solution instead of sterilized Milli-Q water in the assay tubes.

### Determination of INA and freezing behaviour in various tissues

Bark tissues were carefully removed from current-year stems (collected in December) with sterilized razor blades to obtain xylem plus pith and the separated tissues were used for INT determination and differential thermal analysis (DTA). For INA assay, 7.5-mm-long xylem plus pith or bark tissues derived from 7.5-mm-long stems were dispensed into each tube (40 tubes). For DTA, 2-cm-long bark tissue segments and 2-cm-long segments of the xylem with pith were cooled at 5 °C h^−1^ and exothermic events were compared with those of intact stems. In DTA, a cooling rate (5 °C h^−1^) faster than INA assays was employed to avoid any desiccation of the sample during cooling. Experimental conditions of DTA and INA assays were basically the same as for intact stems described earlier.

To check the effect of vacuum infiltration or degassing on the INA of the stem and xylem, samples in some experiments were held in test tubes under vacuum for 5 min to remove air bubbles and fully immersed in water before being assayed for INA.

### Antibiotic treatments of stem samples

When necessary, to check heat stability of the INA in the samples, tubes containing samples previously used for INT determination were autoclaved for 15 min at 121 °C. After discarding leachate, 0.5 mL of sterilized Milli-Q water was added to each tube aseptically prior to determining INT again using the test tube nucleation assay. In experiments using Snomax^®^ solution, the autoclaved solution in each assay tube was not replaced before determining INT. To see the effect of various antimicrobial agents on the INA of the stem, blueberry stems were treated with NaClO (0.1 %, 30 min) and HgCl_2_ (1 %, 1 min). Prior to the determination of INA, the stems were also treated for 30 min, 1 and 2 days at 7 °C with kanamycin sulfate (20 mg mL^−1^), oxytetracycline hydrochloride (20 mg mL^−1^), doxycycline hydrochloride (20 mg mL^−1^), rifampicin (20 mg mL^−1^) and penicillin G potassium salt (20 mg mL^−1^).

### Preparation of various subcellular fractions

To localize INA in the subcellular fractions, bark tissues were excised from current-year stems of high-bush blueberry (cv. Weymouth). Two hundred grams of bark tissues were homogenized with mortar and pestle in 400 mL of 0.5 M mannitol, 20 mM glycylglycine–NaOH (pH 7.5), 5 mM MgCl_2_, 1 mM EDTA and 1 mM DTT. The homogenate was vacuum-filtrated through a 100-μm nylon mesh and two layers of filter paper. The filtrate was sequentially centrifuged at 200*g* for 10 min, 11 000 *g* for 10 min and 100 000 *g* for 1 h to obtain pellet fractions and soluble fraction (supernatant of 100 000 *g*). The soluble fraction was concentrated 50-fold using a centrifugal filter unit (10 000 MW cut off) (Ultrafree-MC, Millipore) followed by dialysis against 20 mM glycylglycine–NaOH (pH 7.5). The debris remaining on the filter papers was rinsed with 1000 mL of the homogenizing medium, followed by 1000 mL of 200 mM NaCl and finally with 1000 mL of Milli-Q water to give the cell wall fraction. For INT determination of each subcellular fraction, ∼20 mg of the cell wall fraction, 50 μL of the homogenate fraction (equivalent to ∼20 mg bark) or 1/200 volume fractions (equivalent to 1 g bark tissues) of the 200 *g* pellet, 11 000 *g* pellet, 100 000 *g* pellet and concentrated soluble fraction were dispensed into each tube (containing 0.5 mL water).

### Infra-red thermography

The freezing process of potted blueberry (cv. Weymouth) plants (5-year-old, 60 cm high, grown under field conditions) in late October (with leaves still attached) was observed with an infra-red thermography camera (TVS-600; Nippon Avionics Ltd). The plants were cooled without artificial ice inoculation in a cold room where the temperature was cooled at 5 °C h^−1^ from 3 to −6 °C. The temperature of the stem at ∼50 cm high was monitored with a thermocouple attached to one of the branches. The experiments were repeated eight times and a typical example is shown (Fig. 3). Images were recorded as analog videos and converted to digital files for image analyses. To reduce background noise, the image frame just before the first freezing event was subtracted from all the subsequent series of image frames to produce referential images (Hacker and Neuner 2008; Yamazaki and Ishikawa 2010). Only thermal images from the plant specimen are shown (Fig. 3).

## Results and Discussion

### INA in various organs of blueberry

To know the distribution of INA, the INTs of current-year stems (7.5 mm long), leaf blades (7.5 mm^2^), flower buds (single whole) and fruits (single entire or half-cut piece) of high-bush (cv. Weymouth) and rabbit-eye (cv. Woodard) were determined. A typical INT determination (i.e. the cumulative percentage of frozen tubes plotted against temperature) of various organs of cv. Weymouth collected in November is shown in Fig. 1B. Sterilized Milli-Q water had a median INT of −18 °C or lower, while the median INT was shifted to −7.2 and −6.3 °C in the presence of leaf samples and flower buds, respectively. Stem samples showed the median INT at −2.3 °C, the highest among the organs collected in November. A similar order in the INA among different organs was observed in other months, which was evident when the median INT data of each organ collected in different months were averaged (Fig. 1A). The highest INA expressed as the median INT was observed in stems (−3.0 °C), followed by flower buds (−6.6 °C), fruits (−7.2 °C) and leaves (−7.3 °C) for cv. Weymouth. In cv. Woodard, the order of median INT (average of samples collected in different months) was identical: stem (−2.5 °C) > flower bud (−6.7 °C) > fruit (−7.4 °C) > leaf (−7.8 °C). The results suggest that the stem had a much higher INT than other organs in both cultivars of blueberry irrespective of the month of sampling. The effect of sample sizes in this comparison was minimal as stem samples longer than 5 mm or leaf blade samples >5 mm^2^ showed similar INT, as detailed below. It was of interest to know how the high INA develops and changes seasonally in blueberry stems, and these were investigated in a separate study (Kishimoto *et al.* 2014).

### Longitudinal distribution of INA along the stem axis

**Figure 2.**
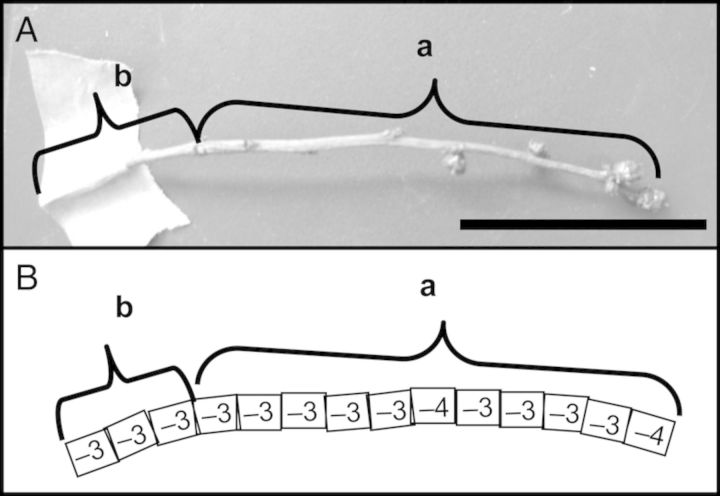
Longitudinal distribution of INA within a 10–15 cm stem of blueberry (cv. Weymouth) in early March. The stem included both current (a) and previous (b) year's growth (A). We determined the INT of every 7.5-mm-long segment from the top to the base (B). The scale bar indicates 5 cm.

Lengthwise distribution of INA in the stem (10–15 cm: current and previous year's growth) of Weymouth in early March was determined by measuring the INA of every 7.5-mm-long segment from the top to the base (Fig. 2). Most of the stem segments had an INA of −3 °C, irrespective of their position along the stem. A similar even distribution was obtained with Woodard stems. This was in agreement with other data of stem INA, showing very narrow variance (Figs 4 and 6, Tables 1 and 2). The results indicated that INA was localized not in specific positions of the stem (nodes/internodes or current/previous-year stem), but evenly throughout the stem. This implies that the stem could initiate freezing at any position spontaneously.

### Freezing process of potted blueberry plants visualized by infra-red thermography

**Figure 3.**
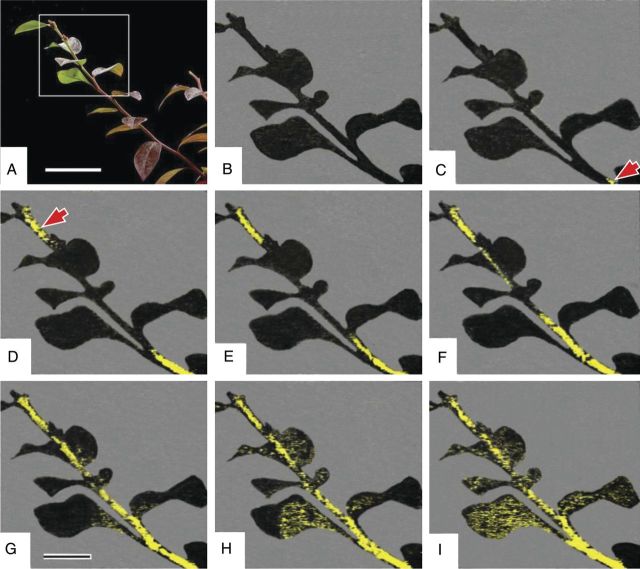
Freezing process of a potted blueberry plant (cv. Weymouth) in late October visualized by infra-red thermography (B–I). The plant used for experiment is shown in (A). Heat release by freezing is depicted in yellow. Freezing initiated in two portions of the stem pointed by the arrowheads (C, D) and propagated in both directions (D–G) and finally into the leaves (G–I). The reference temperature was monitored with a thermocouple. The scale bars represent 5 cm (A) and 2 cm (B–I).

To find how the INA of various organs was related to actual freezing events on whole plants, the freezing process of potted blueberry plants was observed in late October (leaves were still attached) by infra-red thermography. Spontaneous freezing was found to initiate in the upper part and basal part of the stem at −2.5 °C, then to rapidly propagate in both directions in the stem and finally into leaves (Fig. 3). During this process, the soil remained unfrozen and the plants appeared to be dry. Remarkably, the freezing initiated in the stem rather than in leaves attached to the plant, even though the latter had a much larger total surface area and tissue mass. In a separate study, we observed repeated freeze cycles of detached wintering blueberry stems (30 cm long, with no leaves) collected in early March by infra-red thermography (Yamazaki and Ishikawa 2010). Without artificial nucleation, spontaneous freezing initiated at various longitudinal positions on 1- or 2-year-old stems and ice nucleation positions differed even within the same stems from one freeze cycle to another. These observations correspond with the stem having a much higher INA than leaf blades (Fig. 1A) and with the even distribution of INA within the stem (Fig. 2).

### Effect of stem mass on INTs

**Figure 4.**
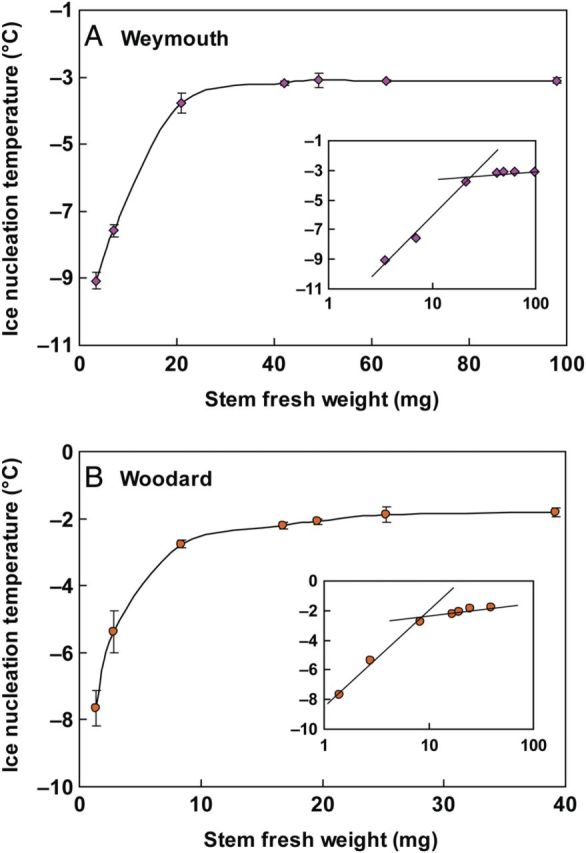
Relationship between ice nucleation temperature (INT) of stems and stem mass determined with two blueberry cultivars, Weymouth (A) and Woodard (B). Current-year stems were collected in early December and used for INT determination using the test tube method. Excised stem samples of 0.83, 1.7, 5, 10, 11.7, 15 and 23 mm in length (weighing 3.5, 7, 21, 42, 49, 63 and 98 mg for Weymouth and 1.4, 2.8, 8.4, 16.8, 19.6, 25.2 and 39.2 mg for Woodard, respectively) were used. Data points represent the mean ± SE of four experiments where the median INT of 40 test tubes containing 0.5 mL sterilized Milli-Q water and samples was determined. Insets are log scale plots of the same data sets. The average stem diameter of Weymouth was 3.2 ± 0.3 mm and that of Woodard, 1.4 ± 0.2 mm.

To clarify the relationship between INT and sample mass, the median INT of blueberry stems collected in December was determined as a function of fresh weight contained in the assay tubes (Fig. 4). The median INT of stems increased from −9.1 to −3.8 °C with increasing stem fresh weights from 3.5 to 21 mg in Weymouth and from −7.7 to −2.8 °C with stem weight increased from 1.4 to 8.4 mg in Woodard. Further increases in stem fresh weight incurred only marginal additional increases in median INT in both cultivars. The 7.5-mm-long stem samples used for routine assays (weighing ∼32 mg for Weymouth and 13 mg for Woodard) represented the INT values in this plateau range (which means that the INT obtained was close to the threshold or saturated INT).

Generally, the INA of an ice nucleator is proportionally correlated to the logarithm of sample concentration at dilute concentrations. However, at higher concentrations, the INA becomes saturated and the median INT approaches a threshold temperature. This holds true with ice-nucleating bacteria and silver iodide when measured in our test tube nucleation assays (data not shown) as well as in droplet nucleation assays (Maki *et al.* 1974; Kozloff *et al.* 1983). This tendency was also evident with the median INT of blueberry stems when plotted in a semi-logarithmic manner (Fig. 4, insets). Increases in the logarithm of stem mass up to ∼25 mg in Weymouth and ∼10 mg in Woodard proportionally shifted the median INT to higher temperatures. When the stem mass was over ∼25 mg in Weymouth and ∼10 mg in Woodard, the stem median INT entered a saturated range as represented by the interpolated lines approaching the horizontal. A similar logarithmic relationship between the median INT and the sample mass was previously reported with INA in *Prunus* wood (Ashworth and Davis 1984; Andrews *et al.* 1986; Gross *et al.* 1988), but they did not describe that at high concentrations the median INT approaches a limit. In stem mass ranging in the order of 10 mg to 1 g (equivalent to 2.3 mm and 23 cm in Weymouth) that are encountered in INT determination experiments, there seems to be a threshold INT (upper limit or saturated value) specific to the stem of each blueberry cultivar.

**Figure 5.**
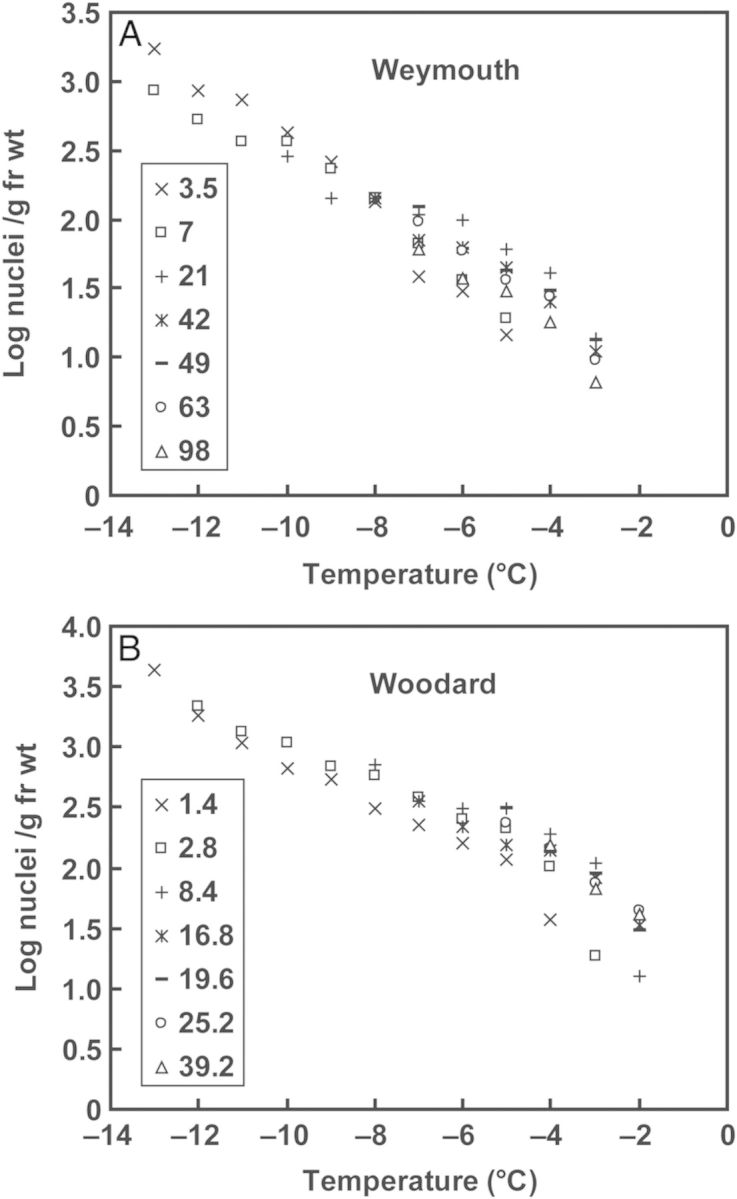
Cumulative ice nucleation spectra of blueberry stems of Weymouth (A) and Woodard (B), showing *K*′(*T*), the concentration of ice nuclei that acted at all temperatures warmer than *T* per unit sample weight. This was calculated from the raw INT data (40 or more replicates for each stem fresh weight represented by different labels ranging from 3.5 to 98 g for Weymouth) shown in Fig. 4 using Eq. (3) as detailed in the Methods section. The ordinate (*y*-axis) shows Log_10_ *K*′(*T*).

In the analysis of heterogeneous ice nucleation in supercooled droplets, Vali (1971) developed a method to obtain the cumulative ice nuclei spectrum, which represents the concentrations of ice nuclei that acted at all temperatures warmer than the selected temperature using the raw data of ice nucleation assays (Eqs (2) and (3) in the Methods section). This was applied to the raw data of our test tube nucleation assays with various mass of stem samples shown in Fig. 4. The cumulative concentration, *K* ′(*T*), of ice nuclei per gram fresh weight sample was calculated using Eq. (3) according to Vali (1995) and shown on a logarithmic scale (Fig. 5). The number of ice nuclei active at −6 °C or warmer was estimated to be 60 per gram stem of Weymouth and 250 per gram stem of Woodard, while those active at −3 °C or warmer were 11 per gram stem of Weymouth and 70 per gram stem of Woodard. More precisely, the stem INA is localized in bark tissues (as detailed later); the ice nuclei concentration can be expressed per gram of bark tissues using the bark/stem ratio. The estimated concentration of ice nuclei active at –3 °C or warmer becomes 37 and 200 per gram bark of Weymouth and Woodard, respectively.

These estimations are valid on the assumption that the model of Vali (1971) fits the nature of ice nuclei of plant origins and the test tube nucleation assays employed in this study. The major concern may be the time-dependent or ‘stochastic’ element of ice nucleation, which is not taken into consideration in Vali's ‘singular’ model (1971). His analysis of time-dependent nucleation (Vali and Stansbury 1966) revealed that the time-dependent nature of nucleation was not seen in samples with high INA (mean INT: −12 °C such as melted hail) but was clearly visible in samples with low INA (mean INT: −18 °C such as melted snow). The following is how this was explained by Vali and Stansbury (1966). Clusters of water molecules in an ice-like configuration formed on heterogeneous nuclei are unstable (easily decay) and undergo fluctuations in size. The mean size of these clusters (embryos) increases with decreasing temperature while the critical size required for the embryos to become ice crystals decreases with decreasing temperature. Therefore, the probability of the mean size of embryos to grow beyond the critical size increases with decreasing temperatures (temperature-dependent nucleation). Fluctuations in cluster size allow more chances for small clusters (much less than average) to reach the critical size with decreasing temperatures and increasing duration of exposure to the temperature (time-dependent nucleation). This is why time-dependent nucleation is more pronounced in samples with low INA (melted snow) or at lower temperatures than those with high INA (melted hail). In the present assay system, slow cooling rates (∼2 °C h^−1^) were used and time-dependent increases in the number of frozen tubes were more typically seen below −12 °C as detailed in the Methods section. Since we are mainly dealing with INT between −1 and −12 °C, the possible error due to time-dependent nucleation is considered to be minimal.

The concentration of ice nuclei in the stem estimated using Vali's model refers to the concentration of ice nuclei which eventually acted and not to the potential concentration of ice-nucleating substances *per se*, which is unknown. As can be deduced from the above discussion, the substance responsible for high INA is most likely to have a large surface to accommodate water clusters as the critical size required to initiate freezing at warmer subzero temperatures is large. It has already been shown that bacterial ice-nucleating sites are large constructs: ice nuclei active at −3 °C are more than 2 × 10^7^ Da and those active at −13 °C, ∼10^5^ Da (Govindarajan and Lindow 1988; Fall and Wolber 1995). A similar situation can be expected in the ice-nucleating substances in blueberry stems. The relationship between the substance concentration and the chances of its going into action can be elucidated only after such substances are isolated.

### Localization of INA within the stem and its relation to DTA profiles of the stem tissues

**Figure 6.**
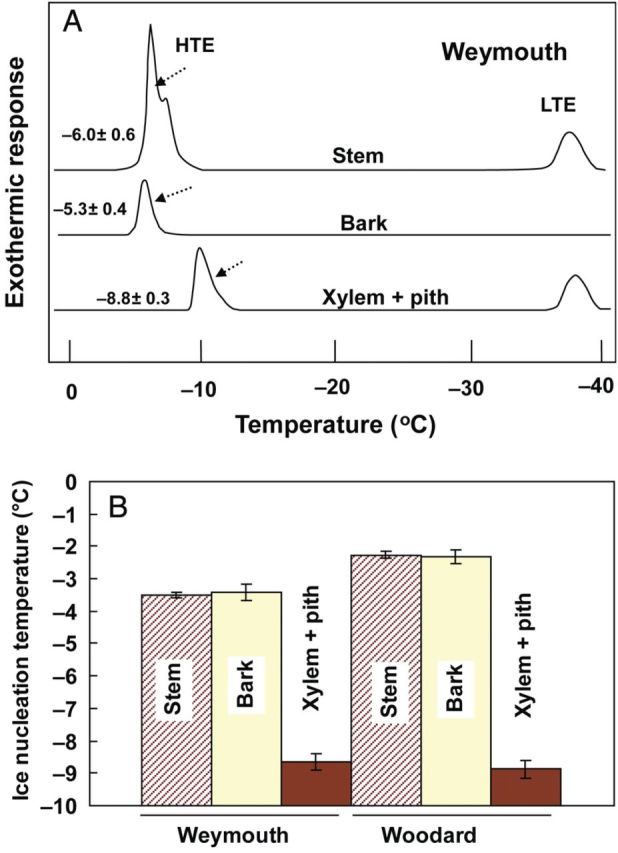
Differential thermal analysis profiles of current-year blueberry stems and their component tissues (cv. Weymouth) collected in mid-December (A) and their corresponding INT determined using the test tube method (B). Bark tissues were carefully removed from the stem with a knife to obtain xylem plus pith and the separated tissues were used for INT determination and DTA. The DTA profiles are typical examples (2 cm long) from six replications (mean ± SE of starting temperature of HTE are indicated). The INT data represent the mean ± SE of four determination of the median INT of 7.5-mm-long segments.

To locate the source of the high INA in blueberry stems (Weymouth in December), bark tissues were excised from the rest of the stem (xylem plus pith) for comparative determination of INA (Fig. 6B). Weymouth stems in December had a median INT of excised bark at −3.4 °C, which was close to that of whole stem (−3.5 °C) while excised xylem plus pith had a much lower median INT (−8.6 °C). Similarly, Woodard stems in December had median INTs of excised bark, whole stem and excised xylem with pith at −2.3, −2.2 and −8.8 °C, respectively (Fig. 6B). Similar results were observed with stems of both cultivars in January (data not shown).

**Figure 7.**
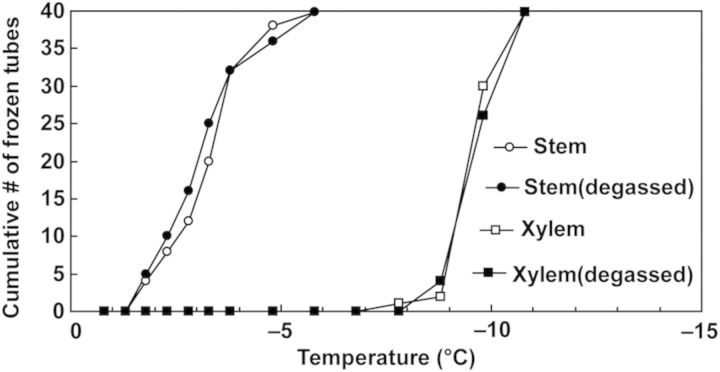
Effect of degassing on INA of excised xylem plus pith segments and the whole stem segments. The samples were used for INA assays with or without degassing treatment by vacuum infiltration for 5 min.

During the process of excision, the excised tissues could dehydrate and it is possible that the INA may not be properly determined as the responsible ice nucleators may not be in direct contact with water. To eliminate this possibility, the excised xylem, bark and the whole stem immersed in water in the test tubes for INA assays were degassed under vacuum for 5 min before starting the ice nucleation assays. Vacuum-infiltration treatments, however, did not significantly influence the INA in any of excised xylem, bark or whole stem (Fig. 7; data of bark tissues not shown). These lines of evidence clearly support the hypothesis that the high INA in blueberry stems is localized in the bark tissues and not in the xylem or pith.

To further substantiate this idea, freezing events in blueberry stems and component tissues were analyzed using DTA (Fig. 6A). A current-year stem piece of Weymouth collected in December had a high temperature exotherm (HTE) starting at around −6 °C with a shoulder peak at −7 °C and a low temperature exotherm (LTE) with a peak at −38 °C. Low temperature exotherm represented the lethal freezing of deep-supercooled xylem ray parenchyma and pith. Excised bark showed an HTE at −5.3 °C and no LTE while excised xylem with pith had an HTE starting at −8.8 °C and an LTE at −38.7 °C. Similarly, Woodard stems in December had an HTE at −6.3 °C, which was close to that of bark (−5.8 °C) and much higher than the HTE of xylem plus pith (−9.5 °C) (data not shown). Differential thermal analysis in this experiment employed a faster cooling rate (5 °C h^−1^) than the test tube INA assays (2 °C h^−1^). This probably shifted the INT (HTE) to lower temperatures than the INT determined in the test tube assays. The HTE of the whole stem and the excised bark occurring at similar temperatures suggests that ice nucleation, detected in terms of the initiation of HTE in the whole stem, arose from the bark.

Both INA assays and DTA results unanimously support the hypothesis that high INA in blueberry stems is assignable to bark tissues. This event is considered eventually to initiate extracellular freezing of the bark tissues (as detected as the HTE peak in DTA). Subsequently, the freezing is most likely transmitted to other tissues (shoulder peak in DTA) in intact stems except for deep-supercooled xylem ray and pith. The water in xylem vessels in intact stems is most likely ice-nucleated from the bark tissues (shoulder peak in DTA at −7 °C) as the median INT of the xylem and pith was low (−8.6 to −9.3 °C in Fig. 6B and 7) and the HTE of the xylem plus pith initiated at around −9 °C (Fig. 6A).

Similar INA differences in the stem tissues were reported with *R. japonicum* by Ishikawa *et al.* (2000) and Ishikawa (2014). Their median INTs were −6.3 °C in the bark, −12.4 °C in the xylem and −12.8 °C in the pith, determined without vacuum infiltration. The cells in the pith of current-year stems of blueberry and *R. japonicum* are alive and have thick cell walls. Interestingly, excised pith only had an LTE below −30 °C and no HTE peak was detected in DTA profiles of *R. japonicum* (Price *et al.* 1997). This suggests that the whole pith remains stably supercooled as low as −30 °C which contrasts with the xylem where only xylem ray parenchyma remain deeply supercooled. Since pith cells do not undergo extracellular freezing but remain deeply supercooled, they do not require high levels of INA outside the cells while nil levels of INA are favoured inside the cells. In this sense, the lack of efficient INA in the pith was in agreement with their intrinsic freezing behaviour (deep-supercooling). Whether the INA levels of −13 °C observed with *R. japonicum* pith are due to contamination during tissue excision or to the native INA of the tissue remains to be investigated.

### Distribution of ice within stem tissues

**Figure 8.**
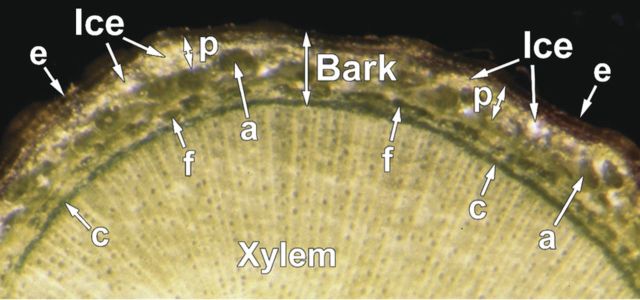
Localization of ice crystals in current-year stems of blueberry (cv. Weymouth collected in late December) cooled at −2 °C h^−1^ to −10 °C and stored for 3–5 days before being observed at −10 °C using a binocular microscope. Ice crystals are shown with arrowheads. e, epidermis; c, cambium; f, fibre; a, aerenchyma; p, cortical parenchyma.

To determine the relationship between INA of stem tissues and localization of ice, we observed blueberry stems (cv. Weymouth sampled in December) slowly cooled and stored at −10 °C for 3–5 days using light microscopy (Fig. 8). Larger ice crystals were mainly localized just beneath the epidermis where cortical parenchyma cells were located and some crystals near the fibres. Much smaller ice crystals were observed in the phloem and cambium tissues while ice in the xylem vessels was not readily visible but the xylem was recognized as frozen by hard texture when pressed with forceps. Cortical parenchyma (not clearly visible as they were buried under ice crystals in Fig. 8), the phloem and cambium tissues were obviously undergoing extracellular freezing. Large air spaces (aerenchyma) in between the cortical parenchyma and the fibres were relatively free of large ice crystals. The aerenchyma may possibly work as a cushion for the accumulated ice crystals in cortical tissues, which may increase in volume within the limiting circumference of the stem.

Accumulation of large ice crystals in the cortex may arise partly from cortical tissues having very large vacuoles even in December. These were likely to be rich in cellular water. It is also possible that such ice localization is less harmful to the plants in terms of mechanical injury due to ice accumulation (Ashworth and Kieft 1995). A high INA in bark may help such extracellular ice formation in specific sites (ice sink) in the tissues by allowing earlier initiation of freezing. Another possible function of the high INA is to retain the ice crystals by promoting recrystallization in the cortex as ice management to avoid freeze desiccation of the tissues. This would be possible if the INA can function in ice nucleation using the vapour phase of water molecules, which remains to be investigated.

### Subcellular localization of INA within the bark tissues

**Figure 9.**
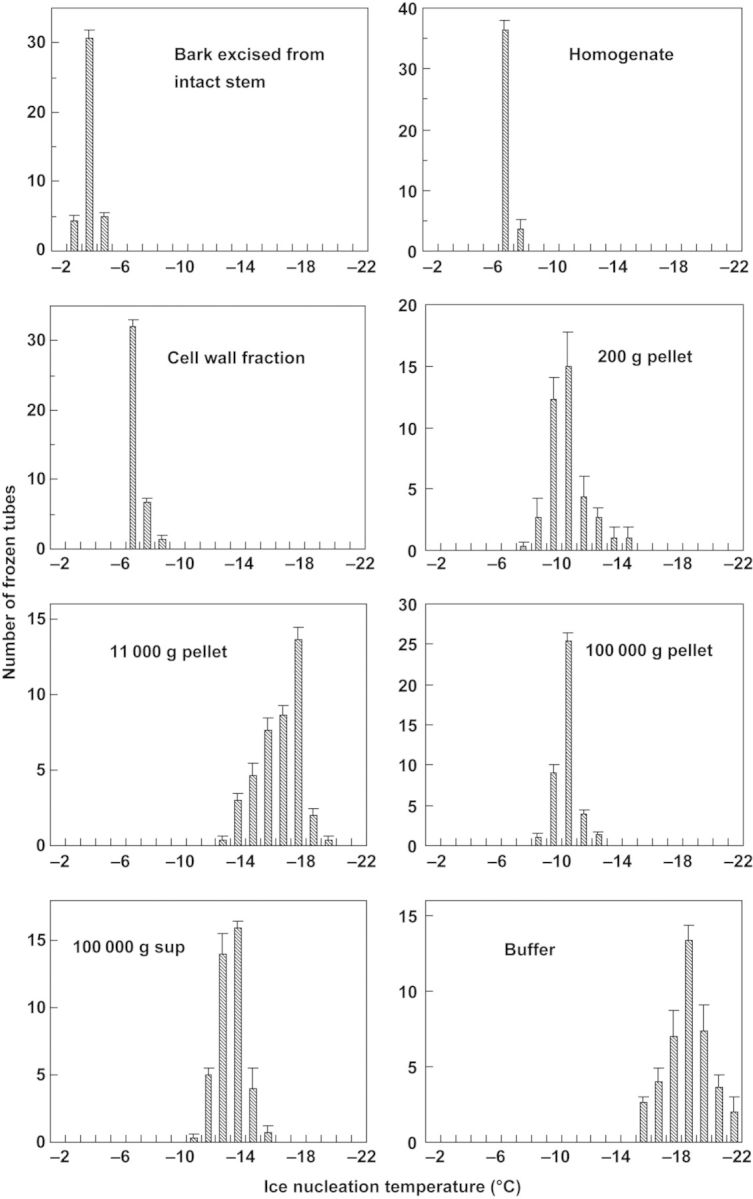
Ice nucleation activity in various subcellular fractions obtained from the bark tissues of blueberry stems (cv. Weymouth collected in January). Excised bark tissues were homogenized in the homogenizing buffer (0.5 M mannitol containing 20 mM glycylglycine–NaOH, pH 7.5) and fractionated by differential centrifugation as described in the Methods section. The INT of each fraction following dialysis was determined using the test tube method and expressed as the temperature distribution of frozen tubes. Data represent the mean ± SE of three determinations.

To investigate subcellular localization of the INA in blueberry stems, bark tissues excised from Weymouth stems in January were homogenized and fractions separated by differential centrifugation were allocated for INA assays. The highest INA (expressed as median INT) was detected in the homogenate (−6.5 °C) and the cell wall fraction (−6.6 °C). Much lower INA was detected, in 200 *g* pellet (nuclear rich −11.4 °C), 11 000 *g* pellet (mitochondria and plastid rich: −16.3 °C), 100 000 *g* pellet (microsomes −10.8 °C) and in soluble fractions (−13.0 °C). The INT histogram of the cell wall fraction showed asymmetric and centralized distribution between −6 and −7 °C, which was similar to that of the homogenate fraction (Fig. 9). This was in contrast to the much wider and more symmetric temperature distribution of INT in other fractions. The cell wall fraction contained mainly cell walls but also a few structural components such as fibres in the bark when observed by microscopy. When the cell wall fraction was further rinsed with 0.5–1 M NaCl to remove ionically bound substances, the high INA was still retained in this fraction (data not shown).

These results suggest that the INA of blueberry bark tissues is localized and tightly bound in the cell walls and/or intercellular structural components. Rationally, the presence of high INA in the cell walls (presumably on their outer surface) and/or neighbouring structural components facilitates ice formation outside the cells or intercellular space in the bark (Fig. 8) at warm subzero temperatures. This allows the cells to undergo extracellular freezing due to the chemical potential difference between the extracellular ice and the intracellular cell sap. The lack of efficient ice nucleators inside the cells likely contributes to retaining the cells in supercooled state until the completion of cell water migration to extracellular ice. The occurrence of INA in a specific tissue and a specific cellular component also implies that the INA in the blueberry stem is more likely of intrinsic origin rather than of microbial origin. If the INA is of microbial origin, a strict control of its localization by the host (blueberry stems) would be required, which is unlikely.

The INT of intact bark tissues of stems collected in January (−4.1 °C) dropped to −6.5 °C when the tissues were homogenized (Fig. 9). The homogenate and cell wall fractions had an INT of approximately −6.5 °C even when the concentration was increased by 10 and 100- fold (data not shown). Similar declines in INA were observed with homogenized ice-nucleating bacteria (Maki *et al.* 1974; Lindow *et al.* 1989) and some plant tissues (Ashworth *et al.* 1985*a*; *Andrews et al.* 1986). In the case of bacterial ice nuclei, 99.9 % of the ice nuclei active at −4 °C or above (large constructs: 10^7^ Da or over) were lost during cell fractionation, while those active at −7 °C or lower (approximately ≤10^6^ Da) were more stable (Maki *et al.* 1974; Govindarajan and Lindow 1988; Lindow *et al.* 1989). The reason for the decline in stem INA during homogenization is not known but, by analogy to the bacterial INA, the intact INA substance may be a large construct that was disrupted by homogenization or by some other mechanism into smaller units, resulting in lowered activity (Burke and Lindow 1990; Mueller *et al.* 1990; Fall and Wolber 1995).

### Effects of various antimicrobial treatments on INA

To see whether the INA in blueberry stems derives from microbial origin, the stability of the INA to various antimicrobial chemicals and antibiotics (Table 1) was examined. The median INT of blueberry stems collected in January was not affected by NaClO (0.1 % 30 min) and HgCl_2_ (1 % 1 min) treatments, which usually damage most fungi and bacteria. Development of molds or growth of microorganisms was not visually detected after incubation of the stems treated with these chemicals at 7 °C for 1 month. Only marginal decreases in the stem INT were detected during 2 days of incubation with various antibiotics effective against Gram-negative bacteria (kanamycin), Gram-positive bacteria (rifampicin, penicillin G) or both (doxycycline, oxytetracycline). When the winter stems were incubated in polyethylene bags at 7 °C without these antibiotics for ≥2 months or more, they were more or less decayed, with molds developing on the stems. This microbial flora had low levels of INA (median INT: −14.2 °C).

These results imply that the INA in blueberry stems is of intrinsic, rather than bacterial, origin and were basically in agreement with the property of INA in *Prunus* stem being resistant to various surface disinfectants (Ashworth and Davis 1984; Gross *et al.* 1988). Most ice-nucleating bacteria occur epiphytically (Lindow 1983), but the possible occurrence of ice nucleation-active microorganisms in internal tissues that could not be easily killed and live in a more symbiotic manner cannot be completely discounted (Hirano and Upper 1995).

When the stem was autoclaved (121 °C for 15 min), the median INT of Weymouth stems declined from −2.3 to −7.3 °C (November) and from −4.2 to −9.2 °C (January) and that of Woodard from −1.8 °C to −9.2 °C (November) and from −4.3 to −9.4 °C (January) (Table 2). Responses of INA to autoclaving differed depending on the organs of blueberry (Fig. 1A). The levels of INA after autoclaving were similar in stems and buds, but were lower in fruits and lowest in leaves. The median INT of *P. syringae* (Snomax^®^) solution declined from −3.7 to −18 ∼ −19 °C after autoclaving (Table 2), which was similar to the level of distilled water (Fig. 1B). To determine the effect of the presence of ice-nucleating bacteria, stem segments were imbibed in the Snomax^®^ solution (with a median INT of −3.7 °C) instead of sterilized Milli-Q water in the test tube nucleation assays (Table 2). The results show that INT levels of November stems of both cultivars were not affected by the bacterial ice nuclei, but January stems had INT levels similar to the bacterial solution. This is because the INT of intact stems in January was lower than that of bacterial solution, thus the INA of bacteria initiated freezing of the tubes. Following autoclaving, INTs of stems imbibed in bacteria solution were very similar to those of the corresponding autoclaved stems in the absence of bacteria, regardless of months of collection or cultivars. The results indicate that when more than one kind of ice-nucleating substance is in the assay tubes, the one with higher INT determines the nucleation of the water. Different behaviour before and after autoclaving of INA between blueberry stems and bacterial solutions suggests the existence of INA sources other than *P. syringae* in intact blueberry stems.

The INA of ice-nucleating fungi (*Fusarium*) is known to be lost completely (median INT<−14 °C) after heating to 70–80 °C (Ashworth and Kieft 1995). Compared with the known INA in fungi, the blueberry stem INA was relatively more heat stable, but not as resistant as the INA in flower bud scales of *R. japonicum*, which was not affected by autoclaving (Ishikawa *et al.* 2000).

## General Discussion

### Spatial localization of INA in blueberry

Among the various terrestrial organs of blueberry plants, current-year stems had the highest levels of INA while leaves had lower INA in both the Woodard and Weymouth cultivars (Fig. 1). Stem segments from different longitudinal positions had equally high levels of INA (Fig. 2). Quantitative relationships between stem mass and INA levels were demonstrated (Figs 4 and 5), which allowed the number of ice nuclei active at −3 °C or warmer per unit tissue mass to be estimated. Careful tissue excision allowed the high INA in the stem to be assigned to the bark tissues while the xylem and pith had much lower levels of INA irrespective of vacuum infiltration (Figs 6 and 7). Subcellular fractionation of homogenized bark tissues revealed that the high INA was localized in the fraction rich with the cell walls and intercellular structural components while the intracellular fractions had low levels of INA (Fig. 9). To our knowledge, this is the first paper to narrow down the localization of INA from the organ level to the subcellular level using a single plant species.

### Functional roles of the high levels of INA in the stem and their likely intrinsic origin

The localization of high levels of INA in the stem corresponded closely to the freezing process of potted plants visualized by infra-red thermography. Thermography showed that freezing initiated at several positions in the stem and propagated into the leaves without external ice nucleation (Fig. 3). A similar freezing pattern was observed thermographically in evergreen azalea cooled artificially without artificial nucleation (Wisniewski *et al.* 1997). The existence of such high INA corroborates observations of blueberry plants spontaneously frozen under dry surface conditions in late autumn in the field (Kishimoto *et al.* 2014) and more general observations that cold-hardy trees initiate freezing at warm subzero temperatures (−0.6 to −3 °C) under field conditions in the absence of external ice nucleators such as frost, snow or frozen soil (Ashworth and Davis 1984, 1986; Ashworth *et al.*1985*b*; Pramsohler *et al.* 2012). It also explains why cold-hardy plant specimens spontaneously freeze without artificial ice inoculation when this is revealed as HTEs at −5 to −6 °C in DTA (Fig. 6A) (e.g. Ishikawa and Sakai 1981; Ashworth *et al.* 1985*a*) and visually detected by nuclear magnetic resonance micro-imaging (Ishikawa *et al.* 1997; Price *el al.* 1997) or infra-red thermography (Wisniewski *et al.* 1997; Carter *et al.* 2001).

However, these observations do not deny the possibility of blueberry plants becoming ice-nucleated by external ice (frost, snow, etc.) in the field. Such plants employ both extrinsic and intrinsic ice nucleation, the latter being ensured by the presence of high INA in the stem (Figs 1 and 2). If the high INA is restricted to nucleating the whole plant or to branches, a highly concentrated and even distribution of INA throughout the stem (Figs 2 and 4) may not be necessary. The high INA was localized in the bark tissues (Figs 6 and 7), where it was assigned to the subcellular fraction rich with cell walls and intercellular structural components. Thus, the high INA most likely allows every part of stem bark tissue to initiate extracellular freezing spontaneously under any cooling or ice nuclei conditions. Extrinsic ice does not necessarily nucleate plants provided the epidermis cuticles are intact (Hacker and Neuner 2008). Excessive supercooling of cold-hardy tissues has been known to lead to increased injuries (lethal intracellular freezing) when ice formation is eventually initiated (Lindstrom and Carter 1983). This is presumably due to exponential increases in the rates of ice crystal growth with increased extent of supercooling (Pruppacher 1967). In the process of cooling of the plant or stem in the field or under experimental conditions, high INA in the cell wall fraction of blueberry bark seemingly acts as a ‘sensing mechanism’ that detects exposure to temperatures slightly <0 °C and spontaneously initiates freezing (ice nucleation) in the extracellular space of the bark tissues at warm subzero temperatures. This mechanism minimizes the danger of excessive supercooling in the absence of extrinsic ice nuclei and allows the bark tissues to undergo extracellular freezing according to the chemical potential difference between the extracellular ice and the intracellular cell sap. We consider this novel theory to be important for understanding plant cold hardiness and to merit further investigation.

The high INA in the bark may also help extracellular ice formation at specific sites (ice sink) in the tissues by allowing earlier initiation of freezing (Fig. 8). It may also help retain the icicles by promoting recrystallization in the cortex as ice management to avoid freeze desiccation of the tissues, which is only hypothetical at the moment.

The stem INA was found to be resistant to various antimicrobial treatments except for autoclaving (Tables 1 and 2). These properties (different from those of microbial INA), the occurrence of the INA in a specific tissue/subcellular component and the reproducible seasonal changes in stem INA during 10 year or more of determination (Kishimoto *et al.* 2014) imply that the INA in blueberry stems is more likely of intrinsic origin rather than of microbial origin.

### Does the first ice nucleation take place in xylem or in bark?

The localization of high INA in the stem bark accords with one report in the literature (Wisniewski *et al.* 1997). This shows freezing being initiated in the cortex of peach stems as visualized by thermography. This is contrary to the generally held view that freezing in trees occurs first in the xylem based on the large total volume of liquid in xylem vessels (Sakai and Larcher 1987). This theory was mainly based on the work by Kitaura (1967) that freezing was propagated through the xylem in mulberry stems in spring. It has also been corroborated by many thermographic observations of freezing process in leaves: rapid propagation of freezing through leaf veins followed by slow spread of freezing in mesophyll cells (e.g. Wisniewski *et al.* 1997; Ball *et al.* 2002; Hacker and Neuner 2008). This encouraged many researchers to believe that ice nucleation occurred in the xylem or vascular tissues irrespective of organ or season. In some cases, ice nucleation sites have been described by thermography (leaf midribs, petiole base, near vascular bundles and phloem fibres) (Ball *et al.* 2002; Hacker and Neuner 2008; Wisniewski *et al.* 2009), which are yet to be confirmed with INA assays for the reasons detailed in the next section.

The nucleation argument simply based on the water volume may not be appropriate as the huge amount (2.3 × 10^312^ kg) of water is theoretically required for nucleation above −10 °C (Franks 1985), and there must be heterogeneous ice nucleators wherever freezing is initiated (Pearce 2001). Therefore, it is important to measure INA of the tissues responsible. However, few previous reports have attempted this except in the case of peach stems (Gross *et al.* 1988), where INA was shown to be equally distributed in outer and inner stem tissues. This is not necessarily in agreement with the cortex being the probable ice nucleation site in peach stems (Wisniewski *et al.* 1997). In contrast, the present results clearly show that the stem INA was localized specifically in the bark (Fig. 6), which corresponds well with the intrinsic freezing behaviour (extracellular freezing) and icicle localization in the bark (Figs 6 and 8). Recent studies using digital thermography revealed that ice nucleation occurs in the bark at numerous positions in blueberry stems, and is then transmitted to the xylem where the freezing is rapidly propagated (manuscript in preparation). Whether this holds true with other plant species will require verification using both precise INA determination and highly sensitive thermographic analysis.

### Comparison of our INA assays with previous methods

We successfully revised test tube methods and developed an INA assay suitable for small plant specimens using test tubes with 0.5 mL Milli-Q water that had been pre-sterilized by autoclaving as detailed in the Methods section. Using this assay, highly reproducible mean INT values were obtained for a wide range of samples having both high and low INA as seen in small SE of the INT values as discussed above.

The INA assay described here is basically similar to the methods described by Ashworth and Davis (1984) and Gross *et al.* (1988), which used 1 and 10–24 mL of water in the tubes, respectively. The major difference was in the median INT for the control (autoclaved water) without specimens: −18.5 to −19.2 °C in our study (Fig. 1B) whereas it was around −11 to −12 °C in their studies. We used 0.5 mL of water in each test tube, but even when the water volume was increased to 2 mL, the median INT remained around −18 °C (Sekozawa *et al.* 2002; Kishimoto *et al.* 2014). The use of glass tubes instead of TPX tubes did not significantly change the INT profiles of the water control, but accompanied the danger of the glass tubes cracking on nucleation (∼1 %). The great difference in the median INT for the water control between ours and those of other researchers may arise from the quality of water, the surface area of the tubes that are in contact with water and/or the method of sterilization. We realize that the covers (transparent film) and dust contribute a low level of background INA. Consequently, all equipment in contact with Milli-Q water used in the assays was pre-autoclaved for 20 min including the tubes, lids, water itself and all the containers and autoclaves themselves. This reduced the background INA efficiently while other ways of sterilization (gas or gamma rays) did not necessarily reduce the background INA. Moreover, all sample handling was confined to a laminar flow chamber to avoid contamination from air-borne ice nuclei. The use of transparent film also allowed frozen tubes to be easily located without touching the tubes, since vibration of tubes at lower temperatures can often nucleate the tubes (Kishimoto *et al.* 2014). These procedures eliminated nucleation above −15 °C of the control tubes with 0.5 mL Milli-Q water (without samples) (Fig. 1B). Consequently, the screening of the tubes that had potential artefacts due to nucleation by the tubes themselves was circumvented, which was an unavoidable step in the INA assays of Ashworth and Davis (1984) and Gross *et al.* (1988).

Ice nucleation of plants has been extensively studied by infra-red thermography, which is a powerful tool that allows visualization of rapid and complex freezing processes (e.g. Wisniewski *et al.* 1997; Ball *et al.* 2002). Ice nucleation events involve probability. Within the limited number of image figures in research papers, it is often difficult to be certain if ice nucleation in the specimen results from coincidental occurrence of an extrinsic ice nucleator or from intrinsic occurrence of INA in the specimen. This has often left thermographic studies at the descriptive level. In contrast, in the present INA determinations, plant tissues were excised and cooled separately in test tubes that were exposed to equal temperatures. Any freezing events above −15 °C detected in the test tube can be assigned to the INA of the sample. Quantitative analyses can be done to evaluate the INA of the plant tissue part (Figs 4 and 5). Responses of INA to various treatments can also be determined (Tables 1 and 2). The method allows us to make a fair determination and comparison of INA of various plant tissue parts, which is otherwise not possible using thermographic methods alone. On the one hand, the method represents a further step to the ultimate identification of the substances responsible for ice nucleation. On the other hand, it has allowed intensive surveys for the natural occurrence of INA in various tissues of over 600 plant species (e.g. Sekozawa *et al.* 2002; Ueda *et al.* 2002; Ishikawa 2014). More importantly, the INA data we report corroborate the freezing events visualized with thermography and magnetic resonance imaging and *vice versa* (e.g. Fig. 3).

## Conclusions

A newly revised test tube assay allowed comparisons of levels of INA in various plant tissues. Using this method, very high INA was found in stems of blueberry. The high INA was mainly localized in the bark where it was tightly bound to the cell wall fraction (cell walls and/or intercellular structural components) while intracellular fractions had little or no INA. The presence of high INA in the bark cell wall fraction corresponded well with the freezing behaviour (extracellular freezing) of bark tissues, ice accumulation in the bark and ice nucleation sites in the plant under dry surface conditions. Stem INA was resistant to various antimicrobial treatments, except for autoclaving. These properties differ from those of microbial INA, and the occurrence of the INA in a specific tissue and a specific cellular component implies that the INA in blueberry stems is of intrinsic rather than of microbial origin. No previous publication has reported the precise localization and quantification of INA in woody plant stems and shown its function to be related to the responses of the tissue and plant to freezing temperatures. The widely held view that the xylem is an important site for ice nucleation is downplayed by these findings.

## Sources of Funding

This study was supported by the Program for Promotion of Basic Research Activities for Innovative Biosciences (1999–2004) from Bio-oriented Technology Research Advancement Institution, Bio-Design Program (2006–08) from Ministry of Agriculture, Forestry and Fisheries and grants-in-aid for scientific research (23380023 and 16380030) from Japan Society for the Promotion of Science to M.I.

## Contributions by the Authors

T.K., Y.S. and M.I. designed the experiments. T.K., H.Y. and A.S. conducted the experiments. T.K., H.Y., H.M. and M.I. analysed the data, produced the figures and tables. M.I. wrote the paper. K.K., W.S.P. and M.I. provided advice/instructions on figure design and significant comments on the manuscript.

## Conflicts of Interest Statement

None declared.
